# Two Committees on Medical Legislation

**Published:** 1890-05

**Authors:** 


### TWO COMMITTEES ON MEDICAL LEGISLATION.

Some misunderstanding has arisen in consequence of the appointment, by the President of the Texas State Medical Association, of a Committee on State Board of Health, with authority to secure the passage of an act to regulate the practice. There is a standing committee,—of which Dr. Cupples is Chairman,—entrusted with this work, and they have recently sent out, through the Secretary's office, a very forcible report. The committee above referred to is a special (and auxilliary) committee, appointed by the President in accordance with the recommendations of the committee who were selected to report on his suggestions, and of which Dr. J. D. Osborn was Chairman. Their special function is to endeavor to secure a Board of Health, and as we understand it, incidentally to aid and cooperate with the standing committee in the matter of a law to regulate the practice; and is not, by any means, intended to supercede or conflict with said committee, The President appointed only the Chairman, Dr. A. M. Douglas, and gave him discretion as to his associates. Dr. Douglas is a member of the State Senate, and as there will doubtless be other members of the State Medical Association in the Legislature, it will be in the power of the Chairman to select with judgment and discrimination, and can secure such men as will have influence; and thus the committee will be able to cooperate with Dr. Cupples' committee to great advantage.

